# Physiological response mechanism of European birch (*Betula pendula* Roth) to PEG-induced drought stress and hydration

**DOI:** 10.3389/fpls.2023.1226456

**Published:** 2023-08-15

**Authors:** Jing Kou, Donghan Yan, Baiting Qin, Qiang Zhou, Chunping Liu, Lijie Zhang

**Affiliations:** ^1^ Key Laboratory of Forest Tree Genetics and Breeding of Liaoning Province, Shenyang Agricultural University, Shenyang, China; ^2^ College of Life Engineering, Shenyang Institute of Technology, Shenyang, China; ^3^ Liaoning Forestry and Grassland Administration, Shenyang, China

**Keywords:** European birch, PEG treatment, drought stress, physiological response mechanism, anatomic structure, electron microscope

## Abstract

Drought stress is also one of the important abiotic factors limiting plant growth and development, and the global temperature is rising year by year, resulting in a dry environment in most terrestrial forests, which will continue to affect the growth, development and reproduction of tree species in forests. European birch(*Betula pendula Roth.*) native to Europe, introduced to the mountains of eastern Liaoning in 1981 (annual precipitation of about 800mm), European birch relative to downy birch (*B. pubescens*)has strong adaptability and drought tolerance and cold tolerance, can grow normally in eastern Liaoning, but it is easy to be affected by drought at the seedling stage and cause death, many arid and semi-arid areas have no introduction and practical application of European birch, and there is less research on the drought resistance of European birch. This study used different concentrations of PEG-6000 treatment to simulate drought stress and clarify the changes of various growth physiological parameters and photosynthetic characteristics of European birch seedlings under drought stress, in order to investigate the physiological response mechanism of European birch under drought stress . This study used different concentrations of PEG-6000 treatment to simulate drought stress and clarify the changes of various growth physiological parameters and photosynthetic characteristics of European birch seedlings under drought stress, in order to investigate the physiological response mechanism of European birch under drought stress. The findings demonstrated that stress duration and increasing PEG concentration had a highly significant impact on the growth traits of European birch seedlings (p<0.01); With increasing stress concentration and stress time, antioxidant enzyme activity, membrane lipid peroxidation, and osmoregulatory substance concentrations increased significantly (p<0.01); With increasing stress concentration and duration, photosynthetic parameters and pigments decreased highly significantly (p<0.01); Under different PEG concentration treatments, the anatomical structure of seedling leaves changed more noticeably; there was a significant effect (p <0.05) on the change in mean stomatal length and a highly significant effect (p<0.01) on the change in mean stomatal structure. The study's findings serve as a foundation for the selection and breeding of new drought-tolerant European birch species, as well as a theoretical underpinning for the use of this species in landscaping and the promotion of new drought-tolerant species in China.

## 1 Introduction

One of the key abiotic factors limiting plant growth and development is drought stress. As annual increases in global temperatures cause most terrestrial forests to experience drought conditions, this factor will continue to have an impact on tree growth, development, and reproduction (Bréda et al., 2006; Semerci et al., 2017; Li C. et al., 2022). Additionally, dry environments can exacerbate abiotic stress in trees, making them more vulnerable to water shortage and death (Ruehr et al., 2016). Drought can also affect forest regeneration (Anderegg, 2015). It has become urgent to select and breed new drought-resistant forest tree species, which is also the key to the successful promotion and application of drought-resistant tree species. In recent years, drought-resistant tree species have been selected, cultivated, and promoted on a large scale to improve the environment of arid and semi-arid regions (Wang et al., 2012; Jacobs et al., 2015; Lu et al., 2018; McDowell et al., 2013).

By responding to their environment in a stressful way, trees are able to develop an antioxidant defense system (Ahmad et al., 2019; Wang et al., 2019). Higher levels of reactive oxygen species are produced to hasten plant senescence and death as a result of drought (Yang et al., 2021; Nadarajah, 2020), and the amount of chlorophyll in plants is also rapidly reduced as a result of stress, which causes a rapid decrease due to slower synthesis or faster decomposition (Lourkisti et al., 2021; Ashraf, 2003). In order to adapt their physiological activities to the water deficit environment, plants activate their own response mechanisms (Ruehr et al., 2014; Berner et al., 2017). This causes a series of reactions to the stressful environment they are exposed to, such as water transfer, synthesis of osmoregulatory substances (Li et al., 2022; Liu et al., 2011; Lei et al., 2006), increased activity of antioxidant enzymes, and expression of drought-resistant genes to mitigate the damage caused by drought stress (Ahmed et al., 2009; Basu et al., 2016; Bhusal et al., 2020; Ameye et al., 2012; Baquedano and Castillo, 2006). Additionally, drought stress has an impact on a plant’s growth traits (Anjum et al., 2011), and changes to the number and size of organelles as well as the structure of plant leaf organelles are also observed (Shao et al., 2016; Oustric et al., 2019).

European birch has strong adaptability, when it is in an arid environment, the relevant hormone conduction signals in the cell response to drought stress will be activated, antioxidant enzyme activity will gradually increase with the degree of drought stress, the corresponding decrease in photosynthesis and the accelerated synthesis of osmoregulatory substances are all positive responses to its adaptation to arid environment, so it can be widely promoted and applied as landscaping ornamental forest, windbreak and sand station, water and soil conservation shelter forest (Tabatskaya et al., 2020); (Giagli et al., 2019). However, at present, there are relatively few studies on European birch and its drought stress, and there is no introduction and practical application of European birch in many arid and semi-arid areas, resulting in restrictions on the development and promotion and application of European birch germplasm resources (Pääkkönen et al., 1998). In order to investigate the growth and stress resistance physiology, photosynthetic properties and leaf anatomical structure, microstructure and ultrastructure characteristics of European birch seedlings after experiencing drought stress and rehydration, and to further clarify a series of physiological response mechanisms, drought stress and rehydration tests were conducted on European birch seedlings using the PEG simulated natural drought method. The study’s findings serve as a foundation for the selection and breeding of new drought-tolerant European birch species, as well as a theoretical underpinning for the use of this species in landscaping and the promotion of new drought-tolerant species in China.

## 2 Materials and methods

### 2.1 Materials

European birch seedlings were obtained using live seed propagation. The seeds were sown in a 4:1:1 perlite: vermiculite autoclaved nutritional substrate before being transferred into a seedling bowl with dimensions of 15 cm 12 cm 13 cm and a consistent soil weight of 400 g. The seedlings were housed in a greenhouse with continuous lighting (12 hours each day), humidity (60°C), and temperature (25°C). The average daily light intensity is 800 μmoL·m^-2^·s^-1^.

### 2.2 Methods

When European birch seedlings reached a height of 20 cm or more, they were examined for PEG-simulated drought stress. 180 European birch seedlings were required for the experiment, which was set up with 6 groups of varying PEG solution concentrations (0%, 5%, 10%, 15%, 20%, and 25%), with the 0% PEG concentration serving as the control group and the other 5 groups as stress treatment groups. Each treatment was replicated three times, using 10 seedlings per replicate. From October to December 2022, the stress experiments were conducted by preparing five concentrations of PEG solution and adding 50 mL daily to the corresponding concentrations of European birch seedling mantle and 50 mL of water in the control group. Changes in seedling growth and leaf morphology were tracked over time with the aid of photographs, and on the 10th and 20th days of the stress experiments, leaves were collected from the same position of each treatment seedling. Experiments with rehydration were carried out after 20 days of stress. After the stress experiment, all treatment groups’ levels of rehydration were restored to those of the control group, and samples were obtained as before at the 10th and 20th days following rehydration.

### 2.3 Growth parameter determination

In the 10d and 20th of the stress experiment and the 10th and 20th of the rehydration experiment, the seedling height, ground diameter and biomass were recorded according to the method of Han et al. (2022), and the root-to-body ratio was calculated. Water content determination: weigh 1.0g of seedling leaves of each of the five treatment groups, dry them in an oven at 80°C for 48h and weigh them again, and the difference between the weight of the leaves before drying and after drying is the leaf water content.

### 2.4 Antioxidant enzyme activity determination

Using mixed samples of leaves collected at the 10th and 20th days after treatment with various doses of PEG solution, at the 10th and 20th days after rehydration, the antioxidant enzyme activity was determined. Weigh about 0.1 g of leaf mix sample and grind the homogenate with 0.05 mol/L precooled phosphoric acid buffer solution under an ice bath.Centrifuge at 15,000 rpm for 15 mins, and the supernatant is an enzyme crude extract. SOD activity was determined at 560 nm using (Giannopolitis and Ries, 1977), POD activity was determined at 470 nm using (Nakano and Asada, 1981), and CAT activity was determined at 240 nm using (Zeng et al., 2019).

### 2.5 Photosynthetic trait determination

Using a portable photosynthesis system (model LI-6400, LI-COR Co., Ltd., Lincoln, NE) to determine the maximum photosynthetic rate by photosynthetic photon flux density(Giannakoula et al., 2021), reference cell[CO_2_] was kept at 420 μmoL·moL^-1^, airflow at 500 moL ·s^-1^ and PPFD at 1600 μmoL· m^-2^· s^-1^, photosynthetic rate (Pn), stomatal conductance (Gs), transpiration rate (Tr), and intercellular CO_2_ concentration (Ci) were determined between 8:00 and 12:00 (Iqbal et al., 2019).

The method of (Wilson et al., 2000) was used to determine the amount of photosynthetic pigment.

### 2.6 Measurement of membrane lipid peroxidation and osmoregulatory substances

Reference was made to the method of (Johnston et al., 2007) in determining the MDA content.

The amount of free proline was calculated using the technique described in (Bates et al., 1973).

### 2.7 Leaf anatomy, stomatal morphology, and organelle observation

After 10 days of treatment with various concentrations of PEG, European birch seedlings had their leaves collected, cut into small pieces measuring 5 mm by 5 mm, fixed with FAA for more than 24 hours, dehydrated with an alcohol gradient, and embedded in paraffin to create sections. The leaf anatomical structures were then examined under a microscope after stress. With a few minor alterations, leaf stomatal morphology observation sample processing was carried out using the Regulus 8100 scanning electron microscope. Stomatal observation was then completed(Raza et al., 2020). With a few minor adjustments, the procedure of (Kong et al., 2013) was used to generate leaf organelle structures. After that, the organelles were seen using a Hitachi TEM system.

### 2.8 Data processing and analysis

All the obtained data were classified statistics and polynomial analysis using Microsoft Excel 2010, and the ANOVA test and correlation analysis was performed by SPSS22.0 software, the significance level was set to p<0.05, the very significance level was set to p<0.01, and all the data in the table were displayed in the form of mean ± standard deviation.

## 3 Results and analysis

### 3.1 Effect of different PEG concentration treatments on growth traits of European birch seedlings

Since drought deprives plants of water, resulting in a decrease in the rate of carbon assimilation of plant leaves, European birch seedlings concentrate most of their energy and nutrient supply in resistance to arid environments, so they can’t provide nutrients for plant growth normally, thereby reducing the growth rate of plants. The growth of seedling height (**Table 1**) and ground diameter (**Table 2**), the accumulation of biomass (**Table 3**) of European birch seedlings will gradually decrease with the increase of stress concentration and the extension of stress time, and the lack of water will make the rhizomes of seedlings continue to grow deeper into the soil to obtain more water for their survival, and the growth limit of seedling height gradually reduces the biomass accumulation in the aboveground part, so drought stress will increase the root-to-head ratio of seedlings (**Table 4**). Different PEG treatment concentrations at the same stress time had significant effects on the seedling height, ground diameter, biomass and root-to-head ratio of seedlings (p < 0.01).

**Table 1:** Effects of different PEG concentrations on the height growth of European brich seedlings.

Treatment concentration	Seedling Height/cm				Relative Increase 20d	Relative Increase 30d	Relative Increase 40d
	10d	20d	30d	40d			
CK	27.73 ± 0.45a	37.56 ± 2.25a	45.67 ± 1.12a	48.33 ± 1.20a	34.36%	64.69%	74.28%
T1	25.63 ± 1.47b	34.53 ± 0.97b	40.50 ± 0.79b	44.21 ± 1.63b	34.72%	58.00%	72.49%
T2	24.17 ± 0.43bc	32.00 ± 1.68c	37.97 ± 0.56c	40.30 ± 0.75c	32.39%	57.09%	66.73%
T3	23.36 ± 0.82cd	29.64 ± 1.42c	33.67 ± 1.85d	38.06 ± 0.49d	26.88%	44.13%	62.29%
T4	22.23 ± 0.73d	25.68 ± 0.41d	30.43 ± 0.76e	33.33 ± 1.84e	15.52%	36.88%	49.93%
T5	22.36 ± 0.70d	23.67 ± 0.85d	28.52 ± 1.21e	32.26 ± 0.35e	5.86%	27.54%	44.27%

Different lowercase letters in the same column indicate highly significant differences between treatment concentrations at the same time (p < 0.01).

CK: 0% PEG concentration; T1: 5% PEG concentration; T2: 10% PEG concentration; T3: 15% PEG concentration; T4: 20% PEG concentration; T5: 25% PEG concentration.

**Table 2:** Effects of different PEG concentrations on the ground diameter growth of European brich.

Treatment concentration	Diameter/mm				Relative Increase 20d	Relative Increase 30d	Relative Increase 40d
	10d	20d	30d	40d			
CK	2.85 ± 0.13a	4.49 ± 0.16a	5.99 ± 0.15a	7.03 ± 0.11a	57.54%	110.17%	146.66%
T1	2.76 ± 0.04ab	4.14 ± 0.11b	5.54 ± 0.11b	6.76 ± 0.23b	50.00%	100.07%	144.92%
T2	2.59 ± 0.07bc	3.74 ± 0.12c	4.86 ± 0.12c	5.85 ± 0.15c	44.40%	87.64%	125.87%
T3	2.58 ± 0.21bc	3.21 ± 0.14d	4.14 ± 0.14d	5.07 ± 0.06d	24.42%	60.47%	96.51%
T4	2.37 ± 0.12cd	2.94 ± 0.09e	3.55 ± 0.17e	4.49 ± 0.13e	24.05%	49.78%	89.45%
T5	2.24 ± 0.07d	2.56 ± 0.07f	3.23 ± 0.16f	4.08 ± 0.04f	14.28%	44.19%	82.14%

Different lowercase letters in the same column indicate highly significant differences in the concentrations of different treatments at the same time (p < 0.01). CK: 0% PEG concentration; T1: 5% PEG concentration; T2: 10% PEG concentration; T3: 15% PEG concentration; T4: 20% PEG concentration; T5: 25% PEG concentration.

**Table 3:** Effect of different concentrations of PEG on the biomass of European birch.

TC	Biomass/g·Plant-1							
	The Aboveground Part/g·Plant-1				The Underground Part/g·Plant-1			
	10D	20D	30D	40D	10D	20D	30D	40D
CK	5.619 ± 0.124a	6.646 ± 0.486a	7.249 ± 0.345a	8.201 ± 0.305a	3.010 ± 0.319b	3.616 ± 0.273a	4.161 ± 0.127a	4.565 ± 0.121a
T1	5.870 ± 0.262a	5.524 ± 0.303b	6.256 ± 0.341b	7.975 ± 0.911a	2.379 ± 0.164d	2.719 ± 0.234b	4.233 ± 0.123a	4.480 ± 0.017a
T2	4.452 ± 0.211b	4.206 ± 0.563c	5.853 ± 0.169b	6.222 ± 0.198b	2.611 ± 0.833cd	2.886 ± 0.034b	2.709 ± 0.287b	4.455 ± 0.106a
T3	4.697 ± 0.214b	2.719 ± 0.155d	3.755 ± 0.236c	5.682 ± 0.141c	3.473 ± 0.107a	2.338 ± 0.155c	2.579 ± 0.081b	3.691 ± 0.148b
T4	3.703 ± 0.236c	2.195 ± 0.170d	2.223 ± 0.127d	4.383 ± 0.188d	2.892 ± 0.013bc	2.029 ± 0.136c	1.352 ± 0.075c	2.637 ± 0.087c
T5	2.793 ± 0.208d	2.181 ± 0.176d	3.119 ± 0.225e	3.871 ± 0.141e	2.426 ± 0.081d	2.275 ± 0.085c	2.492 ± 0.167b	3.200 ± 0.265d

Different lowercase letters in the same column indicate highly significant differences between treatment concentrations at the same time (p < 0.01).TC: treatment time. CK: 0% PEG concentration; T1: 5% PEG concentration; T2: 10% PEG concentration; T3: 15% PEG concentration; T4: 20% PEG concentration; T5: 25% PEG concentration.

**Table 4:** Effects of different PEG concentration treatments on root crown ratio of European birch.

Treatment concentration	Root-Crown Ratio			
	10	20	30	40
CK	0.535 ± 0.04c	0.544 ± 0.005c	0.575 ± 0.037c	0.557 ± 0.035d
TI	0.406 ± 0.03d	0.492 ± 0.043c	0.677 ± 0.028bc	0.563 ± 0.043cd
T2	0.599 ± 0.05c	0.694 ± 0.092b	0.462 ± 0.037d	0.716 ± 0.036b
T3	0.740 ± 0.02b	0.860 ± 0.049a	0.689 ± 0.063b	0.649 ± 0.024bc
T4	0.783 ± 0.05b	0.927 ± 0.091a	0.610 ± 0.060bc	0.602 ± 0.042cd
T5	0.871 ± 0.06a	0.923 ± 0.076a	0.802 ± 0.086a	0.827 ± 0.085a

Different lowercase letters in the same column indicate highly significant differences between treatment concentrations at the same time (p < 0.01). CK: 0% PEG concentration; T1: 5% PEG concentration; T2: 10% PEG concentration; T3: 15% PEG concentration; T4: 20% PEG concentration; T5: 25% PEG concentration.

### 3.2 Effect of different PEG concentration treatments on antioxidant enzyme activities of European birch seedlings

**Figure 1:**
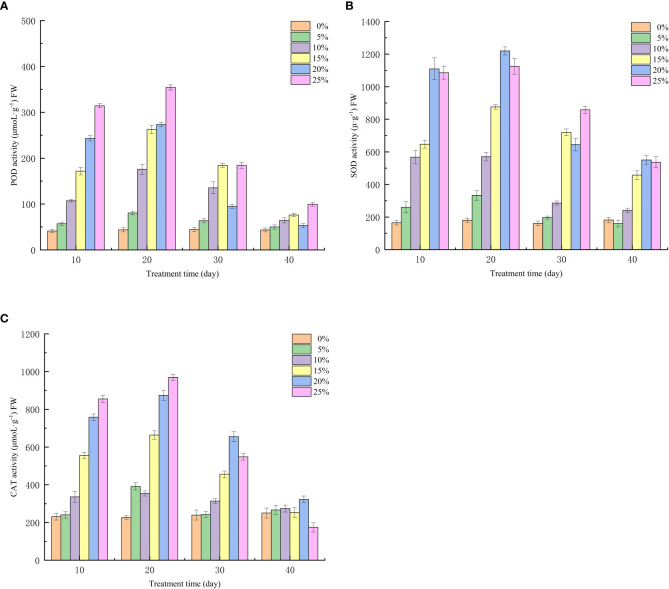
Effect of Different PEG Concentrations on Antioxidant Enzyme Activity of European Betula platyphylla; **(A)** POD, **(B)** SOD, **(C)** CAT.

Drought will lead to a large accumulation of reactive oxygen species in European birch, which is one of the important reasons for plant withering or death, and the strength of antioxidant enzyme activity determines the ability of seedlings to remove free radicals in the body and the reactive oxygen species accumulated due to drought, and the drought stress induced by different concentrations of PEG has a significant effect on the antioxidant enzyme activity of seedlings at different treatment times (p < 0.01), after 10 days of treatment, POD activity increased with the increase of PEG concentration, and the T5 treatment group reached a maximum of 354.29μmoL·g^−1^ at 20 days (**Figure 1A**). Similarly, SOD and CAT activities of all PEG-induced drought-treated seedlings showed an increasing trend at the 10th and 20th days of stress treatment (**Figures 1B, C**). At the 20th day of stress, the SOD activity of seedlings in the T4 group reached a maximum of 1219.89μ·g^−1^. The CAT activity in the T5 treatment group reached a maximum of 968.97 μmoL·g^−1^. It can also be seen from **Figure 1** that the activity of POD and CAT is still on the rise at the 20th day of the highest concentration (25% PEG) in the treatment group, indicating that the seedlings can withstand higher stress concentration and have strong drought resistance. With the rehydration experiment, the seedlings can self-repair a series of problems caused by drought, and the synthesis and accumulation of oxides in the body gradually decrease, so the antioxidant enzyme activity of the seedlings in each treatment group began to decrease on the 10th day after rehydration. At the 20th day of rehydration, the antioxidant enzyme activities of the T5-treated seedlings decreased by 254.79, 794.12 μmoL·g^−1^ and 588.79μ·g^−1^, respectively, compared with the highest.

### 3.3 Effect of different PEG concentration treatments on photosynthetic parameters of European birch seedlings

**Figure 2:**
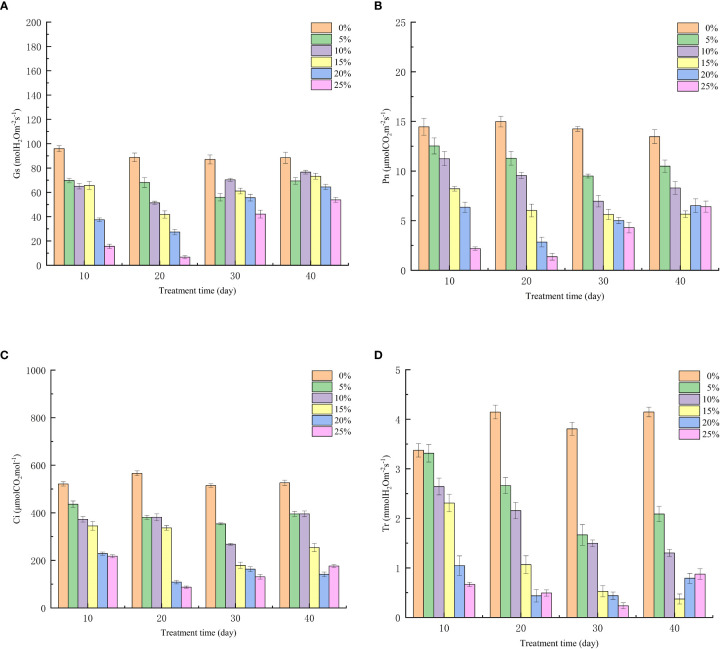
Effect of Different PEG Concentrations on Photosynthetic Parameters of European Betula platyphylla **(A)** Gs, **(B)** Pn, **(C)** Ci, **(D)** Tr.

The change of photosynthetic parameters under drought stress can reflect the regulation and distribution of different links of photosynthesis by seedlings under stress. All organelles in seedling leaves in arid environments will be damaged to varying degrees, and the most important function of leaves is photosynthesis, which makes the photosynthesis of European birch seedlings affected, because the greater the concentration of stress, the more serious the damage to the leaves, so the stomatal conductance (**Figure 2A**), net photosynthetic rate (**Figure 2B**), intercellular CO_2_ concentration (**Figure 2C**) and transpiration rate (**Figure 2D**) will gradually decrease with it, and because the damage to organelles is irreversible, after the rehydration experiment on the seedlings, the photosynthetic parameters were restored to some extent, but not to their original levels. The effects of different PEG concentrations on the photosynthetic parameters of seedlings were very different (p<0.01).

### 3.4 Effect of different PEG concentration treatments on photosynthetic pigments of European birch seedlings

**Figure 3:**
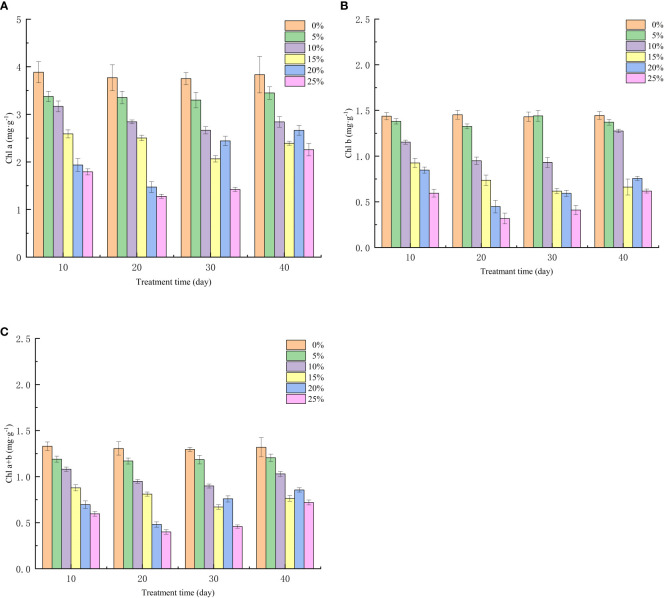
Effect of Different PEG Concentrations on Photosynthetic Pigments of European Betula platyphylla **(A)** Chla, **(B)** chlb, **(C)** chl a+b.

The growth and metabolism of European birch seedlings requires a large number of functional substances, and the accumulation of nutrients is closely related to photosynthesis, photosynthetic pigment is the seedling absorbs light energy and transforms and utilizes it, an important component involved in photosynthesis, the amount of photosynthetic pigment content and the change of content when the seedlings experience stress, determine whether it can provide the key to energy conversion and utilization for plants. The results of this experimental study show that PEG-induced drought stress has a significant effect on the photosynthetic pigment of European birch seedlings (p < 0.01). The content of chlorophyll a (**Figure 3A**), chlorophyll b (**Figure 3B**), and chlorophyll (**Figure 3C**) at different treatment concentrations at the same time, the higher the PEG concentration, the lower the content and the greater the decrease value; It is worth noting that the chlorophyll b content was higher than that in the control group under 5% PEG treatment at the 10th day of rehydration, indicating that mild drought stress could promote the synthesis of chlorophyll b.

### 3.5 Effect of different PEG concentration treatments on membrane lipid peroxidation and osmoregulatory substances in European birch seedlings

**Figure 4:**
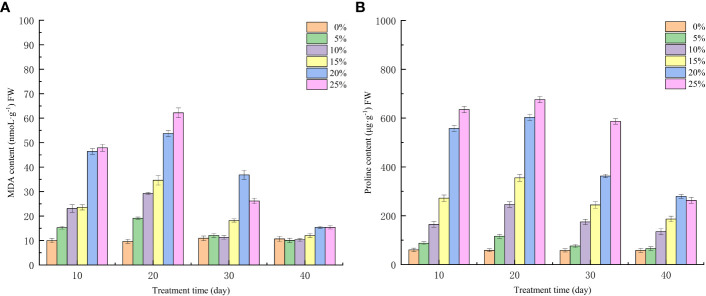
Effects of different PEG concentration on membrane lipid peroxidation and osmoregulatory substances in European birch seedlings **(A) **MDA **(B)** Proline.

Water loss under drought stress will reduce plant cell expansion pressure, osmoregulation ability disorder, resulting in plant death, in this phenomenon, plants evolved to produce a large number of osmoregulatory substances to maintain cell expansion. As observed in **Figure 4**, the effects of drought stress treated with various PEG concentrations were extremely significant (p<0.01) in terms of the changes in MDA and proline levels of seedlings. Proline, an osmoregulatory compound, and malondialdehyde, a result of membrane lipid peroxidation, both of which were elevated following PEG drought stress treatment (**Figures 4A, B**); Except for the control group, where the level of both substances increased with increasing PEG concentration at the 10th day of stress, seedlings in the T4 treatment group had higher MDA content (22.85 nmoL·g^-1^) and higher free proline content (285.98 μg·g^-1^) than those in the T3 group. The free proline content decreased less at 10 days after rehydration but fell to 263.41 μg·g^-1^at 20 days. In contrast, the MDA content of seedlings in the T5 treatment group decreased rapidly after rehydration and stabilized at the 20th day after rehydration in comparison to the control group, approaching the control level.

### 3.6 Effect of different PEG concentration treatments on leaf anatomy of European birch seedlings

**Figure 5:**
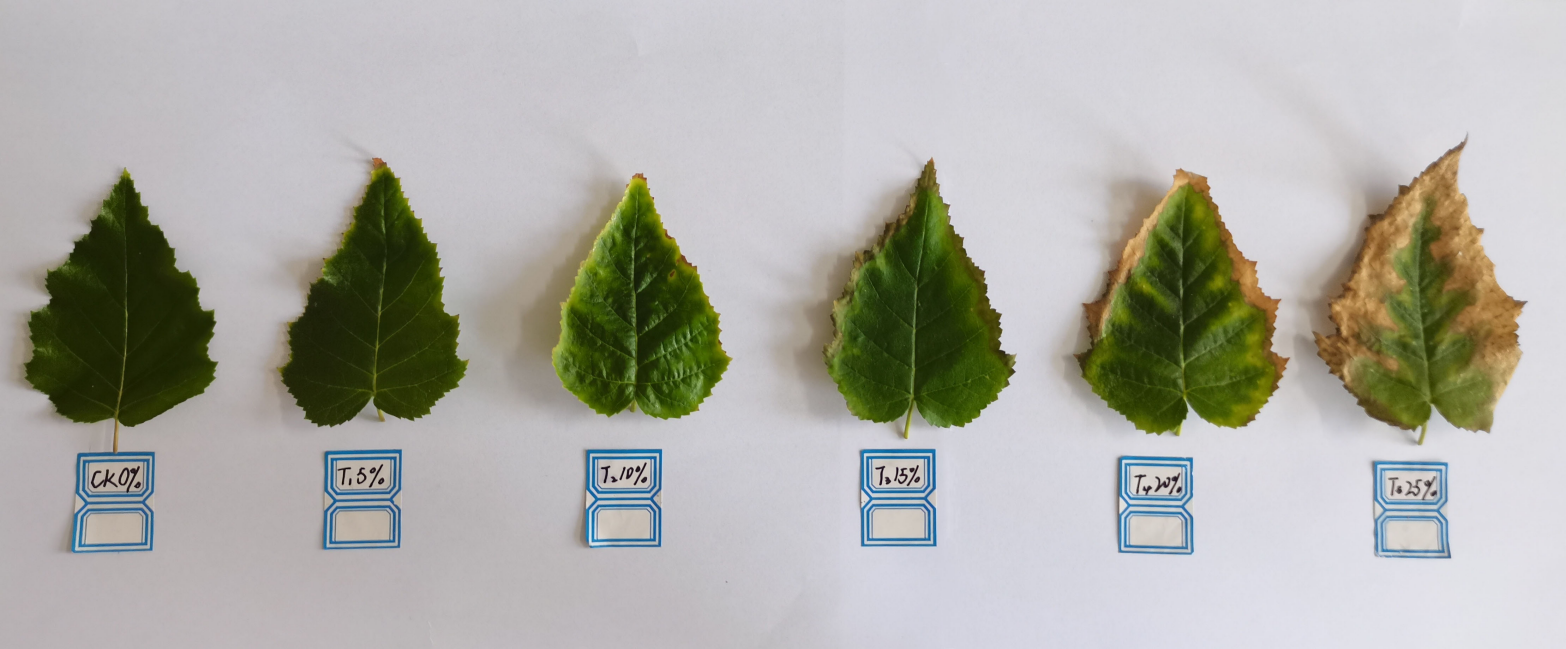
Morphological characteristics of leaves treated with different PEG concentrations for 20 days.

**Figure 5** shows how the leaf shape of European birch seedlings was strongly impacted by various concentrations of PEG drought stress. Compared to the CK group, the seedlings’ leaf color and shape changed after 20 days of PEG treatment. The color of the leaf margin started to lighten in the T2 treatment group, and the leaf veins started to extend around the fold contraction. In the T3 treatment group, the leaf margin started to turn brown, and the leaf color gradually changed from dark to light and spread to the inside. The aforementioned symptoms were more distinct under the higher concentration of PEG treatment, especially the leaves of seedlings in the T5 treatment group, which showed dry and curled near the edge of death near the edge of the fold contraction.

**Figure 6:**
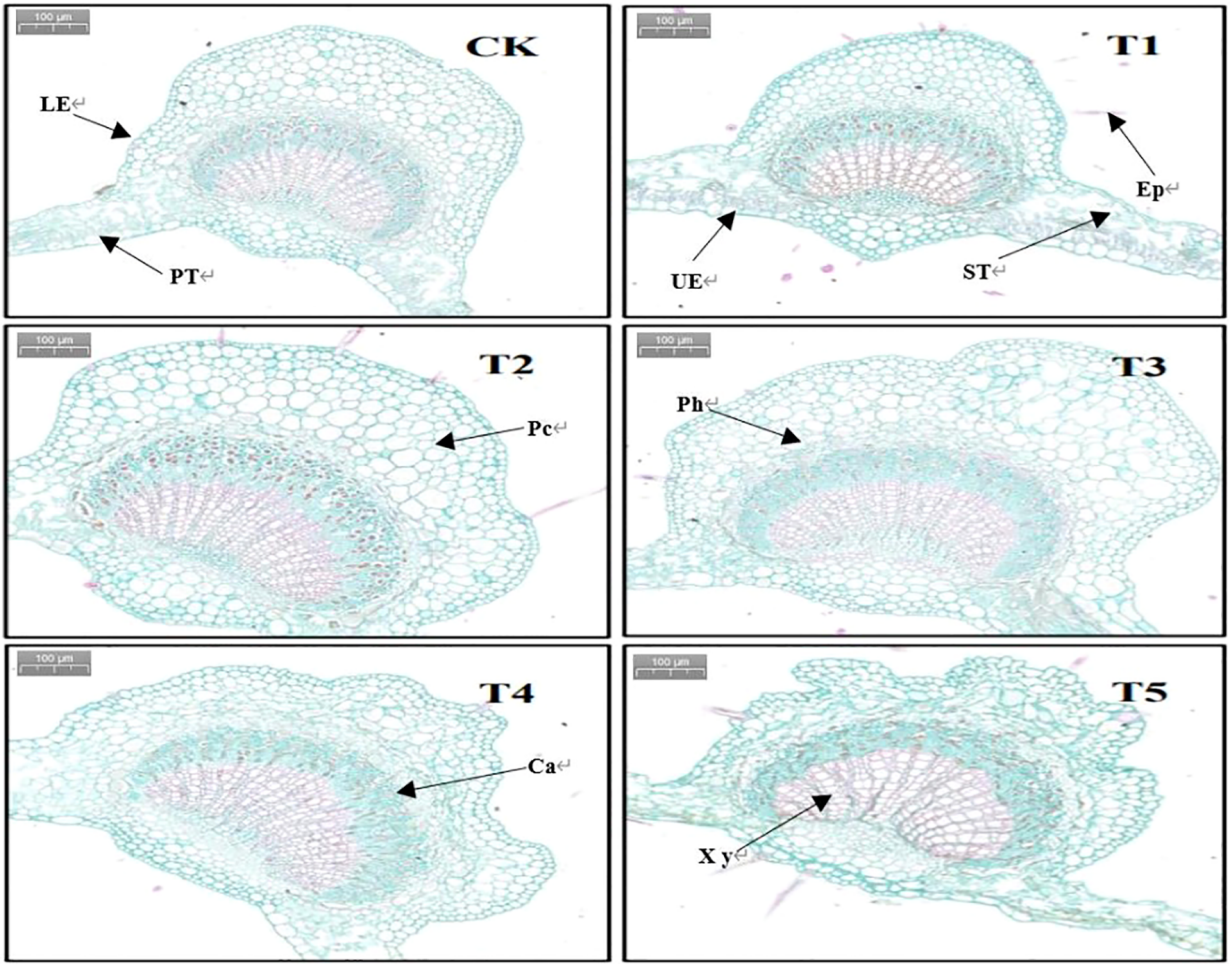
Anatomical structural characteristics of leaves treated with different PEG concentrations for 20 days. UE, upper epidermis; LE, lower epidermis; EP, epifur; PC, parenchymal cells; Ph, phloem; PT, palisade tissue; ST, spongy tissue; Ca, forming layers; Xy, Xylem Section.

The seedling leaf anatomical structure shown in **Figure 6** shows that the epidermal cells were tightly packed and morphologically full and complete in the control group, whereas with an increase in PEG concentration, the leaf cortical cells started to resemble folded sheets, and the morphology of the forming layer cells gradually compressed and stacked, making it difficult to distinguish the forming layer cells in the T5 treatment group.

**Table 5** shows that there was no discernible variation in the ratio of palisade tissue to spongy tissue in the leaves of European birch seedlings among the various PEG concentration treatments. However, the upper epidermal thickness, spongy tissue thickness, leaf thickness, and leaf vein diameter were all significantly decreased by PEG-induced drought stress (p<0.01).

**Table 5:** Changes in leaf anatomical structure parameters after 20 days of treatment with different PEG concentrations.

Anatomical Structure Index	PEG Concentrations					
	CK	T1	T2	T3	T4	T5
UE/μm	25.64 ± 0.86a	23.17 ± 0.53b	20.08 ± 0.78c	19.28 ± 0.48c	16.19 ± 0.26d	13.07 ± 0.65e
LT/μm	11.78 ± 0.35a	10.48 ± 0.58b	9.24 ± 0.45c	7.24 ± 0.53d	5.50 ± 0.44e	4.84 ± 0.20e
PT/μm	22.99 ± 0.61a	21.90 ± 0.76a	19.30 ± 1.21b	17.94 ± 0.43b	15.02 ± 0.70c	13.03 ± 0.84d
ST/μm	46.60 ± 0.73a	42.71 ± 0.54b	37.55 ± 0.96c	35.53 ± 0.72d	30.59 ± 0.84e	28.18 ± 0.68f
P/S/μm	0.49 ± 0.01ab	0.51 ± 0.01a	0.51 ± 0.02a	0.50 ± 0.02ab	0.49 ± 0.03ab	0.46 ± 0.02b
LT/μm	105.25 ± 0.77a	98.38 ± 0.45b	87.94 ± 0.53c	78.92 ± 0.51d	66.64 ± 0.63e	58.72 ± 0.365f
VD/μm	436.90 ± 0.1.47a	426.35 ± 2.46b	406.32 ± 2.02c	376.37 ± 2.00d	340.66 ± 1.54e	310.32 ± 1.03f

UE, upper epidermis; LE, lower epidermis; PT, palisade tissue; ST, spongy tissue; P/S, palisade tissue/spongy tissue; LT, leaf thickness; VD, vein diameter (p < 0.01).

### 3.7 Effect of different PEG concentration treatments on stomatal morphology of European birch seedlings

**Figure 7:**
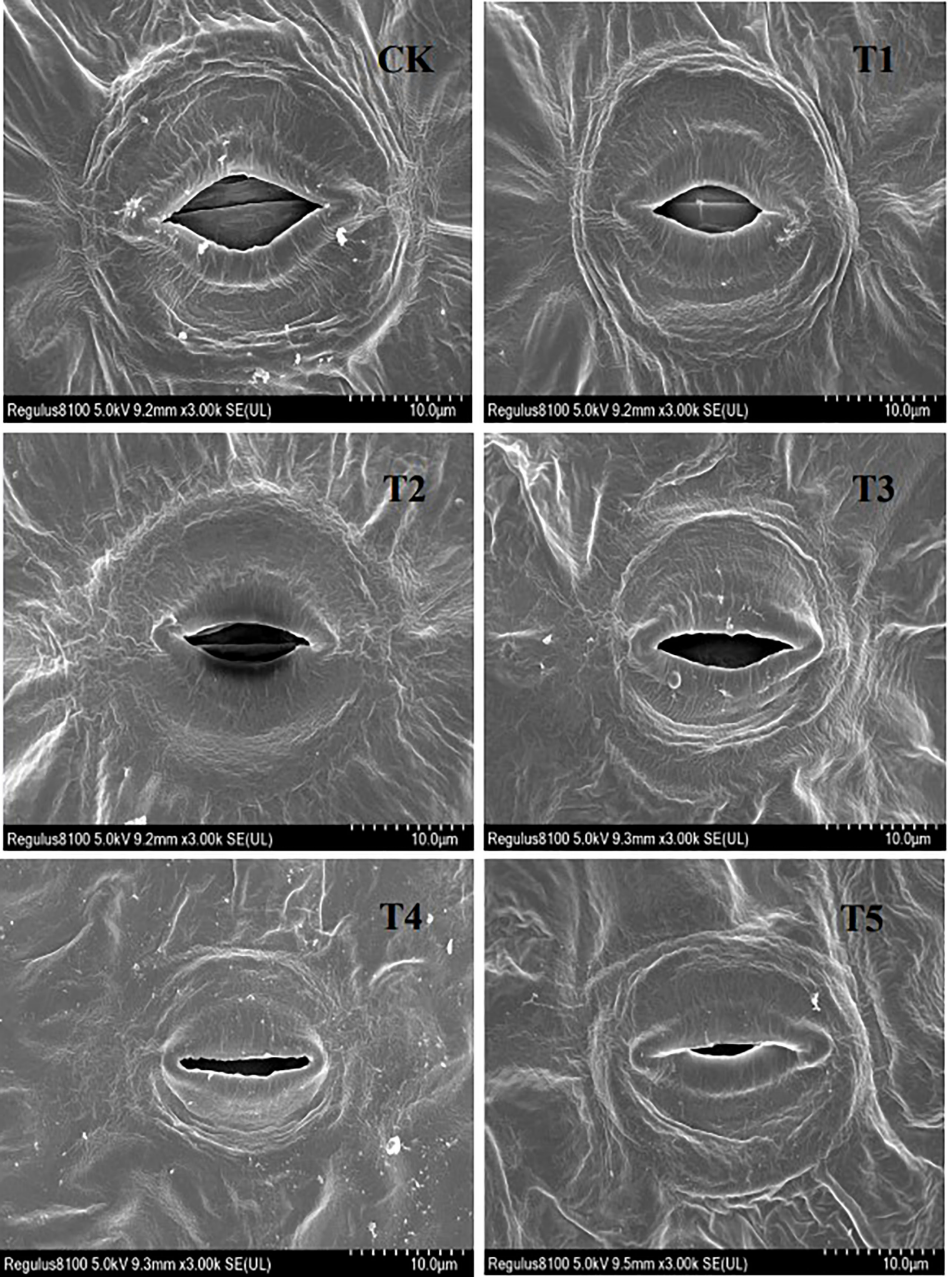
Stomatal characteristics after 20 days of treatment with different PEG concentrations.

**Figure 7** shows how the stomatal characteristics of seedling European birch leaves were affected by different concentrations of PEG-induced drought stress. Stomata only appeared on the seedlings’ lower epidermis, and higher concentrations of PEG treatment led to almost closed stomata in seedling leaves.

In the drought stress environment, the first sensing of plants is water deficit, and most of the flowing water of seedlings will be lost through transpiration, seedlings in order to reduce water loss, will reduce the stomata to open holes or even close, through the statistics and analysis of the change of stomatal structure (**Table 6**), it is concluded that PEG concentration treatment has a significant effect on the average stomatal length between seedling leaves (p < 0.05), and the stomatal width, stomatal pore size and stomatal density are very significant differences (p < 0.01).

**Table 6:** Characteristics of changes in average stomatal structure between leaves after 20 days of treatment with different PEG concentrations.

Treatment concentration	Stomatal Structure			
	Stomatal Length(μm)	Stomatal Width(μm)	Stomatal Aperture(μm)	Stomatal Density(NO·mm^-2^)
CK	44.33 ± 0.75ab	36.53 ± 1.35ab	5.71 ± 0.38a	56.33 ± 1.52e
T1	43.53 ± 0.92ab	37.56 ± 0.70a	5.25 ± 0.41ab	65.00 ± 3.00d
T2	41.60 ± 0.81bc	35.54 ± 1.32b	5.04 ± 0.10bc	75.67 ± 1.53c
T3	44.23 ± 1.42a	36.56 ± 0.94ab	4.72 ± 0.11c	86.67 ± 2.08b
T4	42.6 ± 1.05ab	33.60 ± 0.95c	3.74 ± 0.17d	92.33 ± 1.52a
T5	39.96 ± 1.35c	32.67 ± 0.93c	1.75 ± 0.22e	73.00 ± 1.73c

Stomatal Length (p<0.05); Stomatal Width (p<0.01); Stomatal Aperture (p<0.01); Stomatal Density (p<0.01). CK: 0% PEG concentration; T1: 5% PEG concentration; T2: 10% PEG concentration; T3: 15% PEG concentration; T4: 20% PEG concentration; T5: 25% PEG concentration.

### 3.8 Effect of different PEG concentration treatments on the ultrastructure of leaf organelles of European birch seedlings

**Figure 8:**
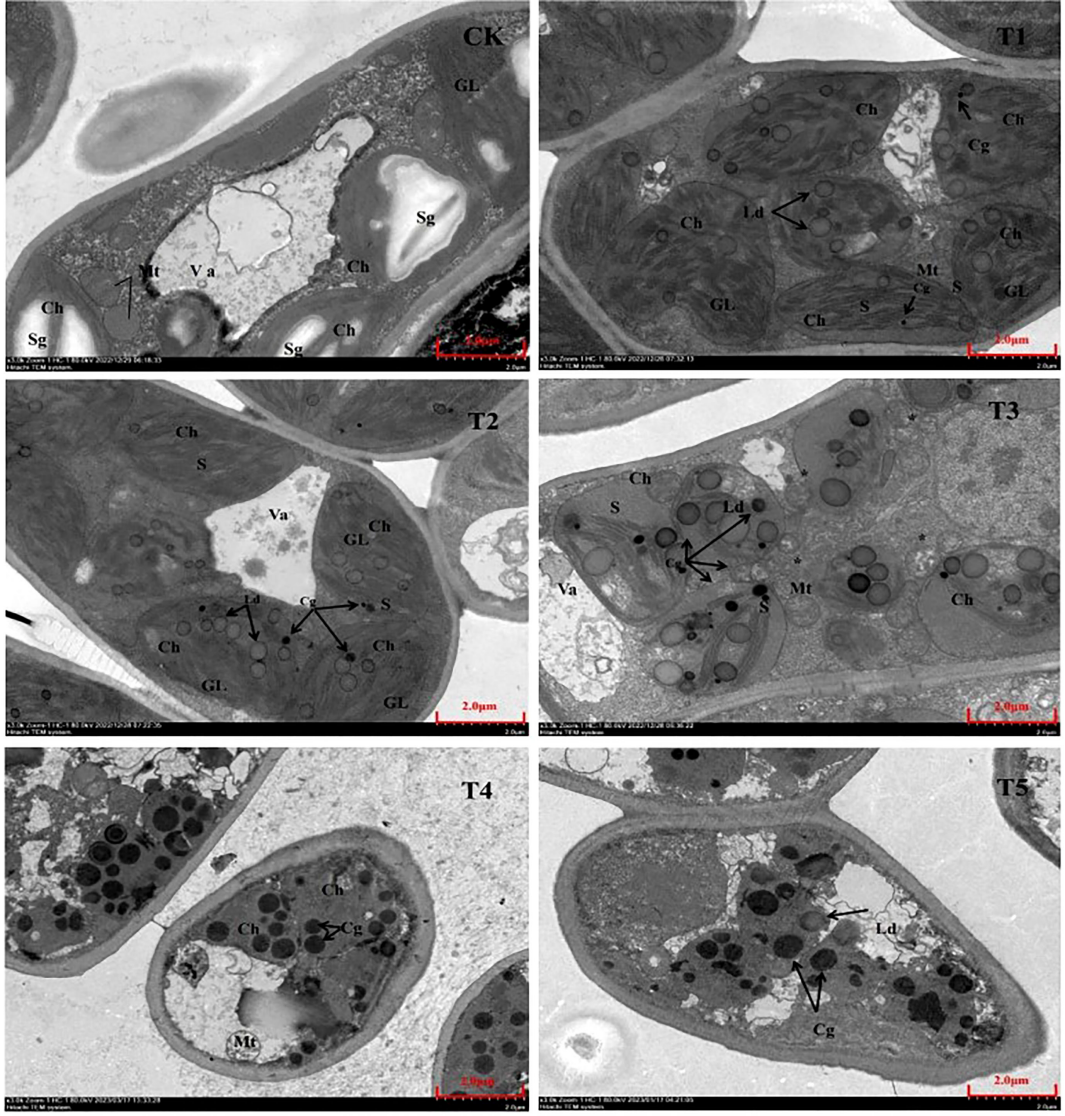
Ultrastructural characteristics of organelle after 20 days of treatment with different PEG concentrations. Va, vesicle; Ch, chloroplast; Sg, starch granule; Mt, mitochondrion; GL, basal lamella Cg, osmium phagocytic granule; Ld, lipid droplet; S, stromal lamella *: cleaved mitochondrion.

Different PEG treatment concentrations have an impact on the shape and quantity of organelles, which are strongly associated to plant growth and metabolism. **Figure 8** shows that the control seedling leaves’ palisade tissue has a complete leaf sarcomere cell morphology and is vesicular-filled; The chloroplasts are larger, flattened or elliptical, and have a distribution that is more vesicle-like, clear morphology, and ordered arrangement inside the chloroplasts; many starch grains can also be observed in the image scattered among the chloroplasts; The morphology of the mitochondria was complete and normal, with a spherical shape. Additionally, it was discovered that with increasing PEG treatment concentrations following PEG drought stress, the chloroplast morphology of seedling leaves gradually altered to a spherical shape. The number of mitochondria increased significantly during the T2 to T3 treatment, and more lipid droplets containing osmium-phagic particles were produced in the chloroplasts. The mitochondria also began to gradually disintegrate from the T3 group, and in the T4 and T5 treatment groups, it was possible to see cell membrane shrinkage, complete lysation of the chloroplasts and mitochondria, blurred intracellular field of view, and no complete organelle structure.

### 3.9 Correlation analysis of antioxidant enzymes and leaf anatomical structure parameters of seedlings

Pearson correlation analysis of antioxidant enzyme activity and leaf anatomical parameters showed that the POD, SOD and CAT activities of seedlings 20 days after stress were significantly negatively correlated with the upper epidermis, lower epidermis, palisade tissue, sponge tissue, leaf thickness and vein diameter of leaves (**Table 7**). Not correlated with palisade tissue and sponge tissue ratio. This indicates that in the anatomical structure of seedling leaves, the leaf loses water and withers thins, and the activity of antioxidant enzymes increases when vascular bundle cells lose water and fold.

**Table 7:** Pearson correlation coefficient between antioxidant enzyme activity and leaf anatomical structure.

Enzyme Activity		Anatomical Structure Index (n=7)					
	UE	LT	PT	ST	P/S	LT	VD
POD	-0.959**	-0.969**	-0.960**	-0.971**	-0.428ns	-0.978**	-0.962**
SOD	-0.936**	-0.975**	-0.945**	-0.966**	-0.366ns	-0.972**	-0.952**
CAT	-0.933**	-0.968**	-0.942**	-0.942**	-0.460ns	-0.969**	-0.977**

** indicates significant at p < 0.01, ns indicates not significant. SOD, superoxide dismutase; POD, peroxidase; CAT, catalase; UE, upper epidermis; LE, lower epidermis; PT, palisade tissue; ST, spongy tissue; P/S, palisade tissue/spongy tissue; LT, leaf thickness; VD, vein diameter.

## 4 Discussion

By analyzing the growth changes of plants during drought stress, it is important to understand the response of plants to water deficit at the whole plant level (Ahmad et al., 2009; Zhang et al., 2004). With increasing drought stress time and PEG treatment concentration, European birch seedling height and ground diameter growth declined in this investigation, which was consistent with the results above. When under drought conditions, plants accumulate a lot of reactive oxygen species in their chloroplasts, which leads to oxidative stress. To counteract this oxidative stress, plants produce a lot of antioxidant enzymes to scavenge reactive oxygen species; as a result, plants with higher antioxidant enzyme induction capacity will be more tolerant to drought (Lum et al., 2014; Rubio et al., 2002; Wang et al., 2009). According to the findings of this study, different PEG concentrations had a significant impact on the three antioxidant enzymes’ activities in European birch seedlings, and those effects increased as PEG concentrations rose. These findings are in line with those of the previous study. The entire process of plant growth, development, and reproduction depends on photosynthesis, which provides the energy for physiological processes and biochemical reactions (Bohnert and Jensen, 1996). Drought stress damages leaves and causes stomatal closure, which inhibits photosynthesis in plants and lowers the values of photosynthetic parameters (Farquhar and Sharkey, 1982). According to the findings of this study, drought stress impairs European birch photosynthesis. As a result, the rate of transpiration, stomatal conductance, intercellular CO_2_ concentration, and leaf net photosynthetic rate are all decreased. Plant photosynthesis depends on photosynthetic pigments, which enable the absorption, transmission, and conversion of light energy (Smirnoff, 1995; Ghobadi et al., 2013). According to the study’s findings, chlorophyll concentration declined as drought stress time and PEG treatment concentration increased. In the higher treatment group, chlorophyll a reduced more than chlorophyll b, which is consistent with the findings above. Under normal circumstances, the production and elimination of intracellular free radicals are in a dynamic equilibrium, but when plant cell bodies undergo peroxidation reactions due to drought stress or other adversities, plant leaf cells are damaged and the permeability of cell membranes increases. MDA is the end product of these peroxidation reactions in plants, and it causes the cross-linking and polymerization of macromolecules (Golldack et al., 2014; Carvalho et al., 2019). Therefore, the change of its content is often used as a study of plant aging physiology and resistance physiology (Xiong and Zhu, 2002). The findings of this study demonstrated that the concentration of free proline, which is the European birch’s response to drought stress through the accumulation of osmoregulatory substances, increased in a stepwise manner with increasing stress time and PEG treatment concentration, and that the content of free proline gradually decreased after rehydration. This change was particularly significant in the high concentration treatment group.

The change of leaf morphology is most obvious when trees experience drought stress, so the change of leaf morphology under different PEG treatment concentrations can reflect the drought resistance of plants to a certain extent (Zhang et al., 2015). Additionally, the results of this study were in line with those of oilseed rape (Zhu et al., 2021), and the three antioxidant enzyme activities were highly significantly negatively correlated with the upper epidermis, lower epidermis, palisade tissue, spongy tissue, leaf thickness, and vein diameter of the leaves (p <0.01). Contrary to the findings of the olive study (Bosabalidis and Kofidis, 2002), the three enzyme activities were not substantially linked with the ratios of palisade and sponge tissue. However, in the current study, leaf thickness gradually decreased with increasing stress concentration, likely because the leaf structure was damaged and could no longer carry out normal physiological metabolism and water uptake activities. A previous study showed that drought stress treatment increased the thickness of all leaf tissues, with the exception of the upper epidermis of the leaf (Ennajeh et al., 2010).However, the majority of drought-tolerant plants exhibit a significant increase in antioxidant enzyme activity as a sign of their high drought tolerance and more active changes in antioxidant enzymes under drought conditions. Studies have shown that plant drought tolerance can vary among species and that the response to drought stress varies among species (Guo et al., 2020).

In recent years, through extensive research on the genetics, genetics and biological and abiotic stress of European birch, European birch has become a model tree species adapted to the environment of northern regions, European birch and down birch are very commercial value tree species in northern Europe, but compared with the down birch, European birch is more suitable for growing in drier areas (Hynynen et al., 2010; Oksanen, 2021), its best habitat is silty soil and fine sandy soil, so European birch has strong drought resistance. This study shows that it is not difficult to reflect its positive response to arid environment from growth physiological response to changes in anatomical structure, stomatal structure and organelle ultrastructure, with extremely high plasticity, and also indicates that European birch has good training ability in arid environment, and the future research on resistance and afforestation technology promotion of European birch can make it better adapt to most areas, bring greater economic value to the local area and lay the foundation for the development of sustainable forestry.

## 5 Conclusion

European birch were all significantly impacted by PEG-induced drought stress (p <0.01) in seedlings’ development, physiology, leaf architecture, and mean stomatal structure. Additionally, European birch adapted to drought stress by slowing down seedling height diameter, biomass, photosynthetic and transpiration rates, leaf thickness, and the ratio of palisade sponge tissue, while also boosting root-to-crown ratio, antioxidant enzyme activity, and free proline content with increasing PEG concentration. Under high PEG treatment, the leaf’s morphology was changed, and Pearson correlation analysis revealed that in European pitcher birches, the relationship between leaf structure and antioxidant enzyme activity was highly significant. The results of this study demonstrated that European birch seedlings’ resistance physiological index values peaked at a PEG concentration of 25%. The seedlings also demonstrated some drought tolerance, and the majority of seedlings were still able to survive normally after receiving rehydration treatment.

## Data availability statement

The original contributions presented in the study are included in the article/supplementary material. Further inquiries can be directed to the corresponding author.

## Author contributions

All authors commented on the manuscript at all stages. LZ and CL conceived and designed the study. JK, DY, BQ and QZ contributed the materials and analysis tools and contributed to the data analysis and manuscript preparation. All authors contributed to the article and approved the submitted version.
